# PAK4 methylation by the methyltransferase SETD6 attenuates cell adhesion

**DOI:** 10.1038/s41598-020-74081-1

**Published:** 2020-10-13

**Authors:** Zlata Vershinin, Michal Feldman, Dan Levy

**Affiliations:** 1 The Shraga Segal Department of Microbiology, Immunology and Genetics, Ben-Gurion University of the Negev, P.O.B. 653, 84105 Be’er-Sheva, Israel; 2 National Institute for Biotechnology in the Negev, Ben-Gurion University of the Negev, P.O.B. 653, 84105 Be’er-Sheva, Israel

**Keywords:** Cell adhesion, Cell signalling, Biochemistry

## Abstract

P21-activated kinase 4 (PAK4), a member of serine/threonine kinases family is over-expressed in numerous cancer tumors and is associated with oncogenic cell proliferation, migration and invasion. Our recent work demonstrated that the SET-domain containing protein 6 (SETD6) interacts with and methylates PAK4 at chromatin in mammalian cells, leading to activation of the Wnt/β-catenin signaling pathway. In our current work, we identified lysine 473 (K473) on PAK4 as the primary methylation site by SETD6. Methylation of PAK4 at K473 activates β-catenin transcriptional activity and inhibits cell adhesion. Specific methylation of PAK4 at K473 also attenuates paxillin localization to focal adhesions leading to overall reduction in adhesion-related features, such as filopodia and actin structures. The altered adhesion of the PAK4 wild-type cells is accompanied with a decrease in the migrative and invasive characteristics of the cells. Taken together, our results suggest that methylation of PAK4 at K473 plays a vital role in the regulation of cell adhesion and migration.

## Introduction

P21-activated kinase 4 (PAK4) belongs to serine/threonine kinases family which is targeted by Cdc42 and Rac, members of small GTPases family^1^. PAK4 is classified in group II of PAKs family, all containing GTPase binding domain (GBD) and a highly conserved kinase domain^2^. PAK4 is vital for embryonic development^2,3^ and is known to regulate actin cytoskeleton organization and other signaling pathways, such as cell migration and growth^4^. PAK4 and other members of PAKs family are frequently upregulated in many cancer tumors^4,5^. Phosphorylation of PAK4 at serine 474 (S474), located at the conserved catalytic domain, is correlated with its activated form^4,6^. PAK4 has a long list of phosphorylation targets, among them are; LIM domain kinase 1 (LIMK1) at threonine 508^4,7^, paxillin at serine 272^8^, Ran at serine 135^9^, BCL2 associated agonist of cell death (BAD) at serine 112^10^ and β-catenin at serine 675^11^. PAK4 positively regulates β-catenin, a key co-activator of T-cell factor/lymphoid enhancer binding factor (TCF/LEF) transcription factors in the canonical Wnt/β-catenin signaling pathway^11,12^. Phosphorylated β-catenin at S675 is more stable and transcriptionally active, sequentially inducing the expression of Wnt-dependent target genes. Moreover, our recent work demonstrated that the SET-domain containing protein 6 (SETD6) interacts and methylates PAK4 at chromatin in mammalian cells, which leads to activation of Wnt/β-catenin signaling pathway. We found that SETD6 is essential for efficient activation of the Wnt/β-catenin-dependent target genes and for the presence of PAK4 and β-catenin proteins at chromatin^13^. Wnt/β-catenin pathway is an evolutionary conserved pathway which governs cell differentiation and growth, cell survival and renewal^12,14,15^. Previous studies have suggested that there is a positive correlation between cell migration and cell adhesion^16,17^, and there are evidences that both are regulated by β-catenin^18^ and PAK4^8,19^. In our current work, we identified lysine 473 (K473) on PAK4 as the primary methylation site by SETD6. Methylation of PAK4 at K473 (K473me) activates β-catenin transcriptional activity. Furthermore, we show that stable expression of PAK4 K473me reduces cell adhesion properties. The altered adhesion of the PAK4 wild-type cells is tightly linked to a decrease in the migration and invasion characteristics of the cells. Dramatic adhesion-related morphological changes were observed in conditions by which PAK4 is methylated, particularly in filopodia structures, actin structures assembly and mislocalization of paxillin to focal adhesions. Collectively, our results suggest that methylation of PAK4 at K473 by SETD6 tunes the adhesion, migration and invasion properties of breast cancer cells.

## Results

### SETD6 methylates PAK4 at lysine 473 in-vitro and in cells

**Figure 1:**
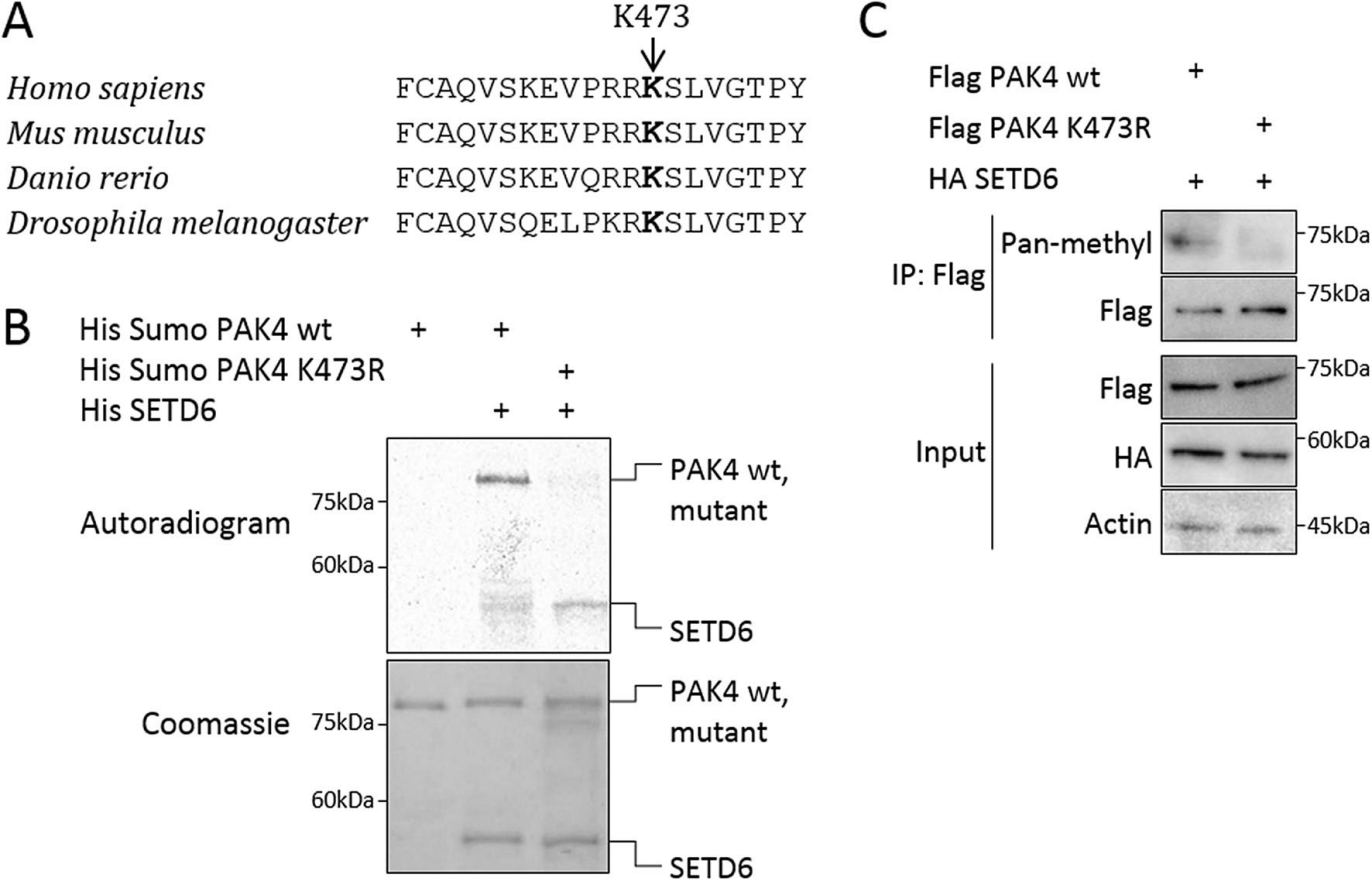
SETD6 methylates PAK4 at lysine 473. (**A**) A multiple alignment of lysine 473 residue of PAK4 in different organisms. Multiple alignment was performed using COBALT tool^55^ for *Homo sapiens*, *Mus musculus*, *Danio rerio* and *Drosophila melanogaster* PAK4 protein sequences. (**B**) In-vitro methylation assay. Recombinant His-Sumo-PAK4 wild-type (wt) or the His-Sumo-PAK4 K473R mutant were incubated with or without His-SETD6 in the presence of ^3^H-labeled SAM. Proteins were then subjected to SDS-PAGE followed by exposure to autoradiogram to detect ^3^H-labeled proteins or Coomassie staining to detect all proteins. (**C**) Methylation assay in cells. MDA-MB-231 wild-type cells were transfected with Flag PAK4 wild-type or Flag PAK4 K473R, and both with HA SETD6 plasmids. Cell lysates were immunoprecipitated (IP) with FLAG-M2 beads, and proteins in IP and input samples were detected by Western blot with indicated antibodies. Methylation was detected with pan-methyl antibody. Uncropped gels are shown in Supplementary Fig. S9.

To map PAK4 methylation site, we mutated several lysine residues in PAK4 protein that are highly conserved among 4 different organisms (*Homo sapiens*, *Mus musculus*, *Danio rerio* and *Drosophila melanogaster*): lysine 31, lysine 51, lysine 350, lysine 442 and lysine 473 (Supplementary Fig. S1A and Fig. 1A). In addition, we mutated lysine 78, which was identified before to be methylated in a proteomic screen at protein modification database PhosphoSitePlus (https://www.phosphosite.org)^20^. The purified recombinant mutants or wild-type PAK4 proteins were subjected to *in-vitro* methylation assay with recombinant SETD6 (Supplementary Fig. S1B). Out of the different mutants that were tested, only the PAK4 K473R mutant showed a significant and repeatable decrease in methylation signal by SETD6 (Fig. 1B). In these methylation assays (Fig. 1B and Supplementary Fig. S1B) SETD6 was auto-methylated, which is consistent with our previous knowledge describing the enzymatic activity of SETD6^21–23^. We tested the methylation of PAK4 K473R mutant also in cells, using a pan-methyl antibody that identified methylated wild-type Flag PAK4 but not the K473R mutant (Fig. 1C). Together, these data suggest that SETD6 primarily methylates PAK4 at lysine 473 in-vitro and in cells.

### Methylated PAK4 at lysine 473 upregulates β-catenin protein levels and Wnt/β-catenin target genes

**Figure 2:**
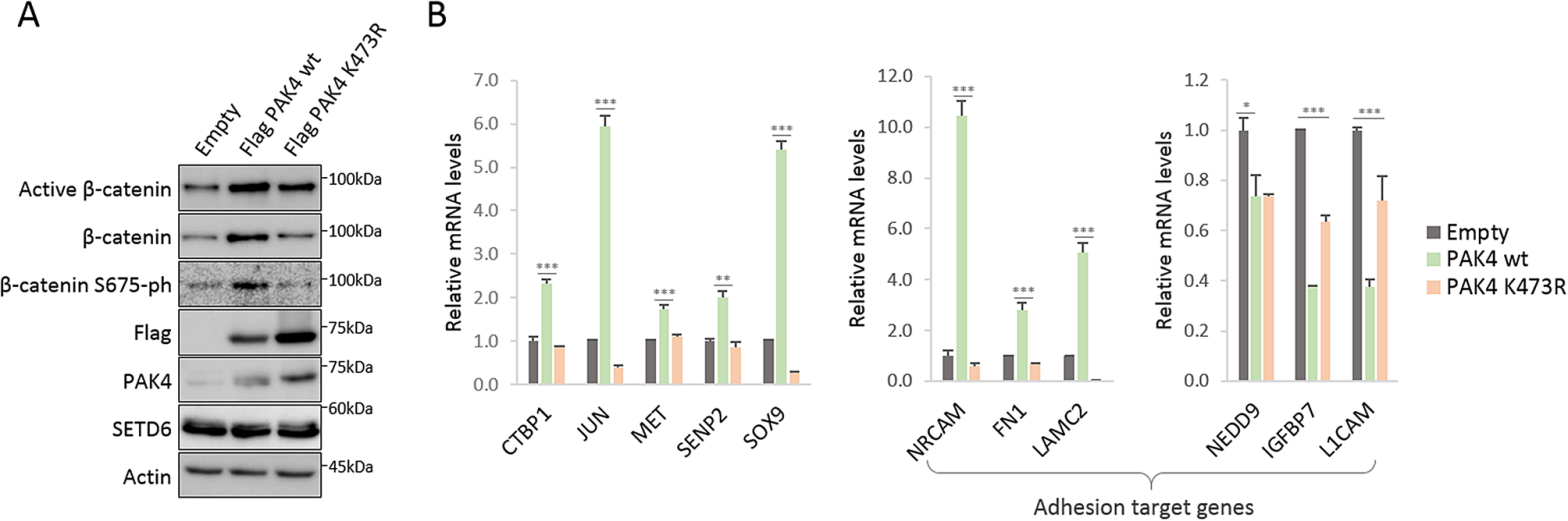
Methylated PAK4 at lysine 473 upregulates Wnt/β-catenin target genes. (**A**) Cell extracts of MDA-MB-231 cells stably expressing empty plasmid, Flag PAK4 wild-type or Flag PAK4 K473R mutant were subjected to Western blot to detect endogenous protein levels of β-catenin, active (non-phospho) β-catenin and β-catenin S675-ph. Uncropped gels are shown in Supplementary Fig. S9. (**B**) mRNA was extracted from MDA-MB-231 cells stably expressing empty plasmid, Flag PAK4 wild-type or Flag PAK4 K473R. Transcript levels of the indicated Wnt/β-catenin target genes were determined by qPCR. mRNA levels were normalized to GAPDH and then to empty cells. Error bars are s.e.m. Statistical analysis was performed for 3 experimental repeats using one-way ANOVA. *p < 0.05, **p < 0.01, ***p < 0.001.

Based on these data and our previous findings^13^, we hypothesized that the methylation of PAK4 at K473 mediates the activation of β-catenin. To test this hypothesis, we generated MDA-MB-231 cells stably expressing Flag PAK4 wild-type or Flag PAK4 K473R mutant that cannot be methylated by SETD6 (Fig. 2A). Our results demonstrate that β-catenin is upregulated (total and active forms) in the presence of wild-type but not the K473R mutant in MDA-MB-231. A reduction in the β-catenin S675 phosphorylation signal was also noted upon stable over-expression of the PAK4 K473R mutant. Consistent with these findings, we performed a quantitative FACS analysis in MDA-MB-231 cells and found that active β-catenin level was increased in PAK4 wild-type, but not in PAK4 K473R stably expressing cells (Supplementary Fig. S2A). Furthermore, isolation of the chromatin fraction revealed that the level of active β-catenin at chromatin was elevated in cells stably expressing PAK4 wild-type compare to PAK4 K473R (Supplementary Fig. S2B), suggesting a direct regulation of gene target expression. In order to test whether these findings are specific to MDA-MB-231 cells, we examined these phenomena in the hormone dependent (estrogen and progesterone) breast adenocarcinoma cell line MCF-7 (Supplementary Fig. S3A). Our previous findings indicate that depletion of SETD6 correlates with a significant reduction in the expression of some known Wnt/β-catenin target genes^13^. We therefore tested the expression levels of Wnt/β-catenin target genes by qPCR in MDA-MB-231 and MCF-7 cells. Our results demonstrate that while the expression levels of Wnt/β-catenin target genes were elevated in PAK4 wild-type cells, no change or a decrease in their expression was observed in MDA-MB-231 cells stably expressing PAK4 K473R mutant (Fig. 2B). We noted significant changes in the expression of Wnt cell-adhesion-related genes. The expression of *NRCAM*^24^, *FN1*^25^ and *LAMC2*^25^ genes was significantly upregulated in cells stably expressing wild-type PAK4 and their expression levels were reduced to a basal degree in the K473R mutant cells, in which PAK4 is not methylated by SETD6. The reduced mRNA levels of *IGFBP7*^26^ and *L1CAM*^25^ following stable expression of wild-type PAK4 expressing cells, were partly restored in cells stably expressing mutant PAK4 (Fig. 2B). We did not see significant changes in the expression level of most of the genes in the control MCF-7 stable cells (Supplementary Fig. S3B). Taken together, our data suggest that specific methylation of PAK4 at K473 orchestrates the expression of the Wnt/β-catenin target genes, among them adhesion-related genes. These findings may also indicate that PAK4 methylation plays a role in the regulation of cell adhesion process.

### PAK4 methylation at K473 decreases cells adhesion

**Figure 3:**
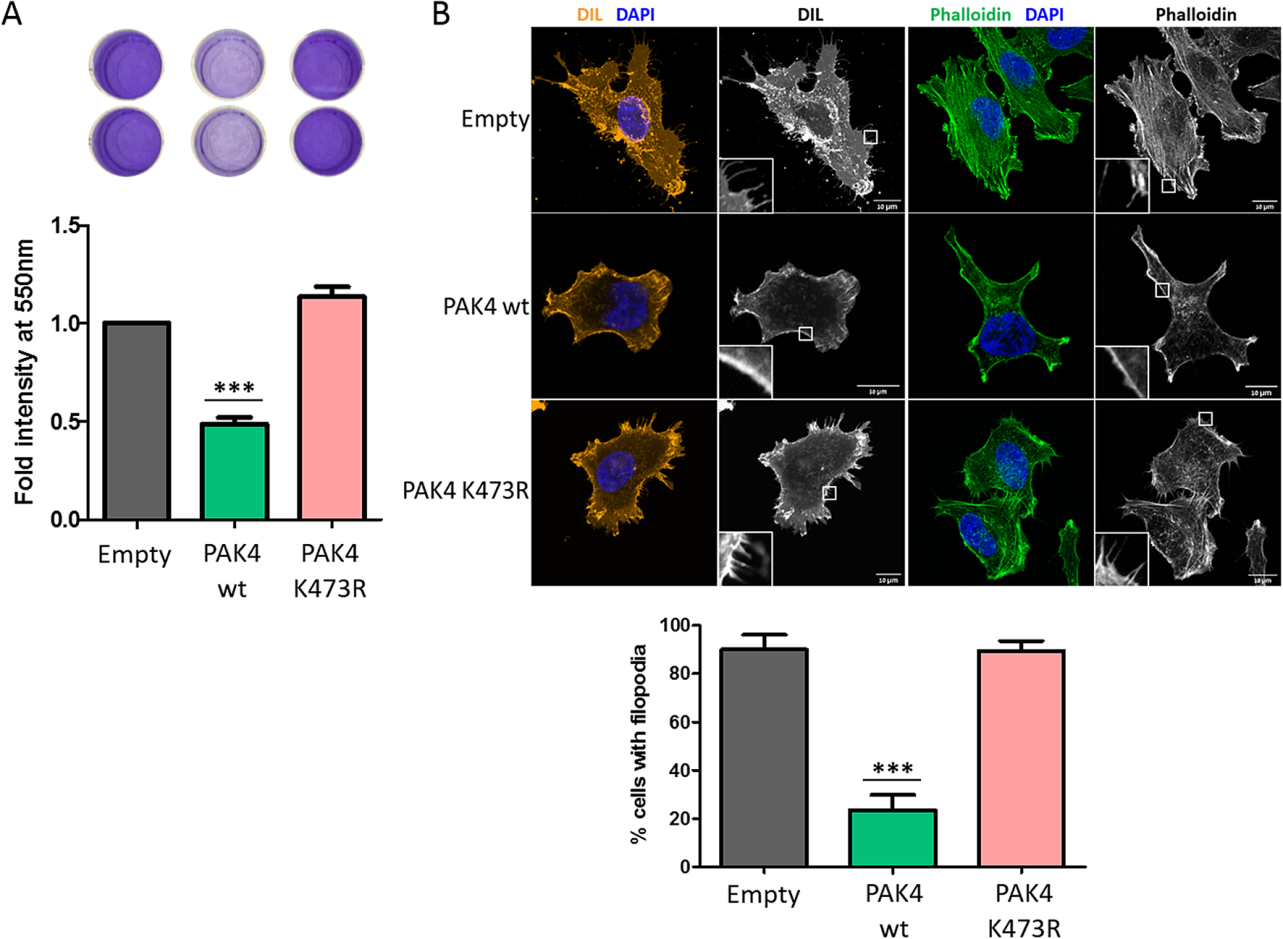
PAK4 K473me decreases adhesion of the cells and modulates cell morphology. (**A**) Cell adhesion to fibronectin. MDA-MB-231 cells stably expressing empty plasmid, Flag PAK4 wild-type or Flag PAK4 K473R were serum starved over-night. Cells were then plated on a fibronectin pre-coated 96-well plate, followed by crystal violet staining. Crystal violet staining was solubilized and quantified. Error bars are s.d. Statistical analysis was performed for 5 experimental repeats using one-way ANOVA. ***p < 0.001. (**B**) Filopodia and cell actin structures staining. MDA-MB-231 cells stably expressing empty plasmid, Flag PAK4 wild-type or Flag PAK4 K473R were fixed and stained with Vybrant™ DiI Cell-Labeling Solution (on the left) or phalloidin (on the right) and Hoechst or DAPI. Cells were then visualized by confocal microscopy (63×). Scale bar 10 µm. Graph on the bottom shows percent cells with filopodia manually counted. Error bars are s.d. Statistical analysis was performed for n > 32 cells per condition using one-way ANOVA. ***p < 0.001.

Both PAK4 and β-catenin were linked before to the regulation of cell adhesion^8,25^. We therefore hypothesized that the methylation of PAK4 at K473 by SETD6 might be involved in the regulation of cell adhesion. A first indication for that link came from a set of experiments where we noticed that cells stably expressing PAK4 wild-type were easily detached from the cell culture plates upon serum starvation. Using crystal violet staining we observed that MDA-MB-231 cells expressing a methylateable PAK4 were easily washed from the plate surface, in contrast to cells where PAK4 is not methylated (stable PAK4 K473R or SETD6 knock-out cells), which were relatively well anchored to the surface (Supplementary Fig. S4). Consistent with these observations, cells stably expressing Flag PAK4 wild-type demonstrated a significant loss of binding to fibronectin-coated plates, in comparison to empty or Flag PAK4 K473R expressing MDA-MB-231 cells which remained adherent to the fibronectin-covered surface of the plates (Fig. 3A).

### PAK4 methylation at K473 diminishes the formation of filopodia and actin structures

Having demonstrated that PAK4 methylation at K473 negatively regulates cell adhesion, we next examined the morphology of the cells using Vybrant™ DiI Cell-Labeling Solution (staining cell membranes) and Hoechst (staining DNA and nuclei) followed by visualization by confocal microscopy (Fig. 3B, left panel). While clear filopodia structures were observed in empty and PAK4 K473R cells, the PAK4 wild-type cells displayed a smooth membrane staining suggesting reduced adhesive properties^27^. Consistent with these results, the formation of actin structures—a feature tightly linked with cell adhesion^28^ and visualized using phalloidin (F-Actin) staining—was noted in cells stably expressing empty plasmid and the mutant PAK4 (Fig. 3B, right panel). Taking together, these results suggest that PAK4 methylation at K473 leads to reduction in cell adhesion and to altered adhesion-related structures.

**Figure 4:**
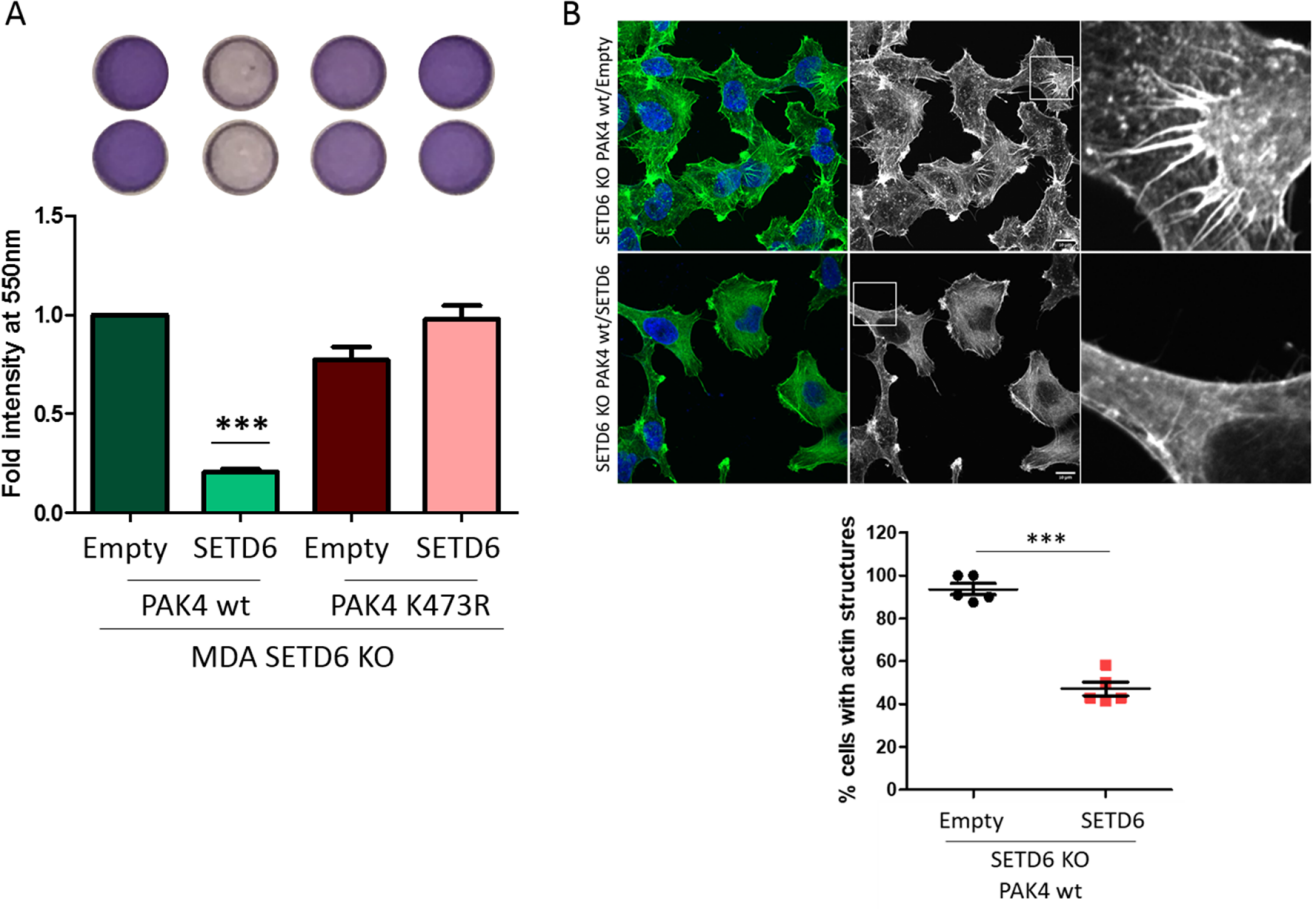
Cell adhesion properties are SETD6 dependent. (**A**) Cell adhesion to fibronectin. MDA-MB-231 CRISPR SETD6 knock-out (MDA SETD6 KO) cells stably expressing Flag PAK4 wild-type or Flag PAK4 K473R with empty or Flag SETD6 were serum starved over-night. Cells were then plated on a fibronectin pre-coated 96-well plate, followed by crystal violet staining. Crystal violet staining was solubilized and quantified. Error bars are s.d. Statistical analysis was performed for 3 experimental repeats using one-way ANOVA. ***p < 0.001, significance for PAK4 wild-type with Flag SETD6 compared to all other conditions. (**B**) Actin structures staining. MDA-MB-231 CRISPR SETD6 knock-out (SETD6 KO) cells stably expressing Flag PAK4 wild-type with empty or Flag SETD6, were fixed and stained with phalloidin and DAPI and visualized by a confocal microscope (40×). Scale bar 10 µm. Graph on the bottom shows percent cells with actin structures manually counted. Error bars are s.d. Statistical analysis was performed for n > 49 cells per condition using Student’s t-test. ***p < 0.001.

In order to verify that the observed phenotypes are SETD6 dependent, we performed a rescue experiment using MDA-MB-231 CRISPR SETD6 knock-out cells stably expressing Flag PAK4 wild-type or Flag PAK4 K473R in the presence or absence of stably expressed Flag SETD6 complementation (Supplementary Fig. S5A shows the protein expression levels). The fibronectin-dependent cell adhesion was lost in the PAK4 wild-type expressing cells when SETD6 is restored in these cells (Fig. 4A). As expected, the cells expressing PAK4 K473R remained adherent, with or without SETD6 (Fig. 4A). Consistent with these results, we could identify a significant decrease in actin and filopodia structures in the cells upon the re-addition of SETD6 (Fig. 4B and Supplementary Fig. S5B). In addition, rescue with SETD6 led to upregulation of Wnt/β-catenin target genes in cells stably expressing PAK4 wild-type but not in cells expressing PAK4 K473R mutant (Supplementary Fig. S5C). Collectively, these data suggest that PAK4 methylation at K473 by SETD6 negatively regulates cell adhesion morphologies.

### PAK4 K473me attenuates paxillin localization to focal adhesions

**Figure 5:**
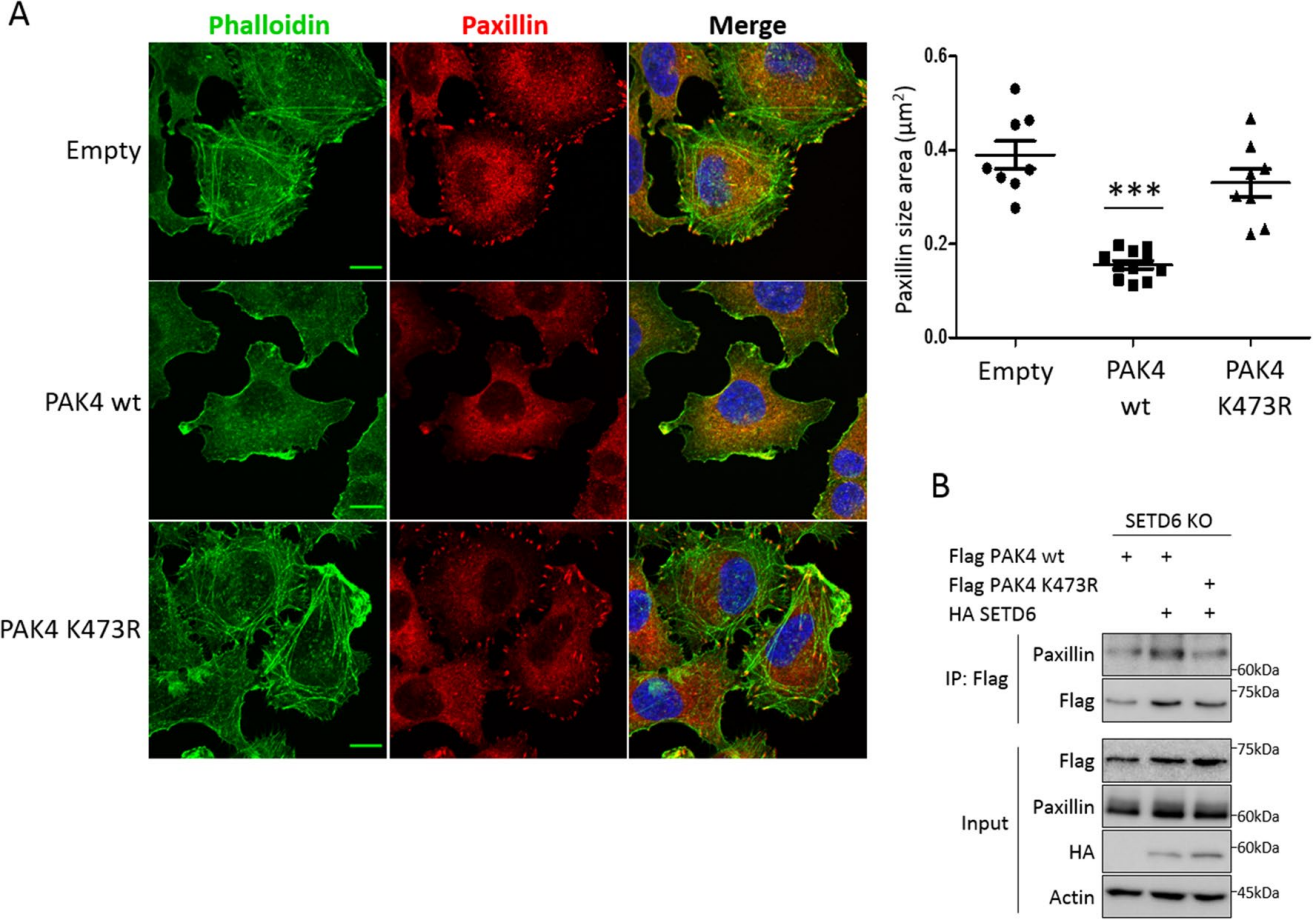
PAK4 K473me attenuates paxillin localization to focal adhesions. (**A**) MDA-MB-231 cells stably expressing empty plasmid, Flag PAK4 wild-type or Flag PAK4 K473R were fixed and stained with phalloidin (green), paxillin (red) and DAPI. Cells were visualized by a confocal microscope (63×). Scale bar 10 µm. Graph on the right represents the paxillin area size. Error bars are s.d. Statistical analysis was performed for n > 44 cells per condition using one-way ANOVA. ***p < 0.001. (**B**) MDA-MB-231 CRISPR SETD6 knock-out (SETD6 KO) cells were transfected with Flag PAK4 wild-type or Flag PAK4 K473R, with or without HA SETD6. Samples were immunoprecipitated with FLAG-M2 beads and submitted to Western blot with anti-paxillin antibody. Uncropped gels are shown in Supplementary Fig. S9.

The assembly of actin structures, such as actin protrusions as well as actin stress fibers, is a cellular feature involved in the generation of focal adhesions^28^, the latter required for cell attachment, spreading and migration^29^. Paxillin, a main component of focal adhesions, was previously shown to be phosphorylated at serine 272 (S272) and activated by PAK4 to stimulate the disassembly of focal adhesions in cells^8,19^. Moreover, it was demonstrated that knock-down of PAK4 in MDA-MB-231 cells resulted in higher cell adhesion to fibronectin due to a lower turnover rate of focal adhesions (greater number and size of focal adhesions in the cells)^19^. We therefore postulated that PAK4 methylation by SETD6 at K473 will attenuate paxillin localization to focal adhesions, leading to defects in focal adhesion assembly. To test this hypothesis, we examined the localization of paxillin by immunofluorescence in MDA-MB-231 cells stably expressing empty, Flag PAK4 wild-type or Flag PAK4 K473R mutant. As shown in Fig. 5A, we found that while in empty and PAK4 K473R mutant cells, paxillin is accumulated at focal adhesions at the periphery of the cells, it is less present on the actin structures in the PAK4 wild-type cells. Consistent with these findings, cells stably expressing Flag PAK4 wild-type exhibited elevated levels of paxillin S272-ph than empty or Flag PAK4 K473R cells (Supplementary Fig. S6A). Overall paxillin protein levels in cells stably expressing empty, Flag PAK4 wild-type or Flag PAK4 K473R, remained unaffected (Supplementary Fig. S6B). We next hypothesized that the methylation of PAK4 by SETD6 may affect the physical interaction between PAK4 and paxillin. The interaction observed between over-expressed Flag PAK4 wild-type and endogenous paxillin in MDA-MB-231 SETD6 knock-out cells, was enhanced upon over-expression of HA SETD6 (Fig. 5B). This increased binding was observed only with Flag PAK4 wild-type and not with Flag PAK4 K473R mutant (Fig. 5B, compare lanes 2 and 3). Taken together, these results suggest that methylation of PAK4 by SETD6 at K473 enhances the interaction between PAK4 and paxillin and attenuates paxillin localization to focal adhesions, leading to the disassembly of focal adhesions in cells.

### PAK4 K473me inhibits cell migration and invasion

**Figure 6:**
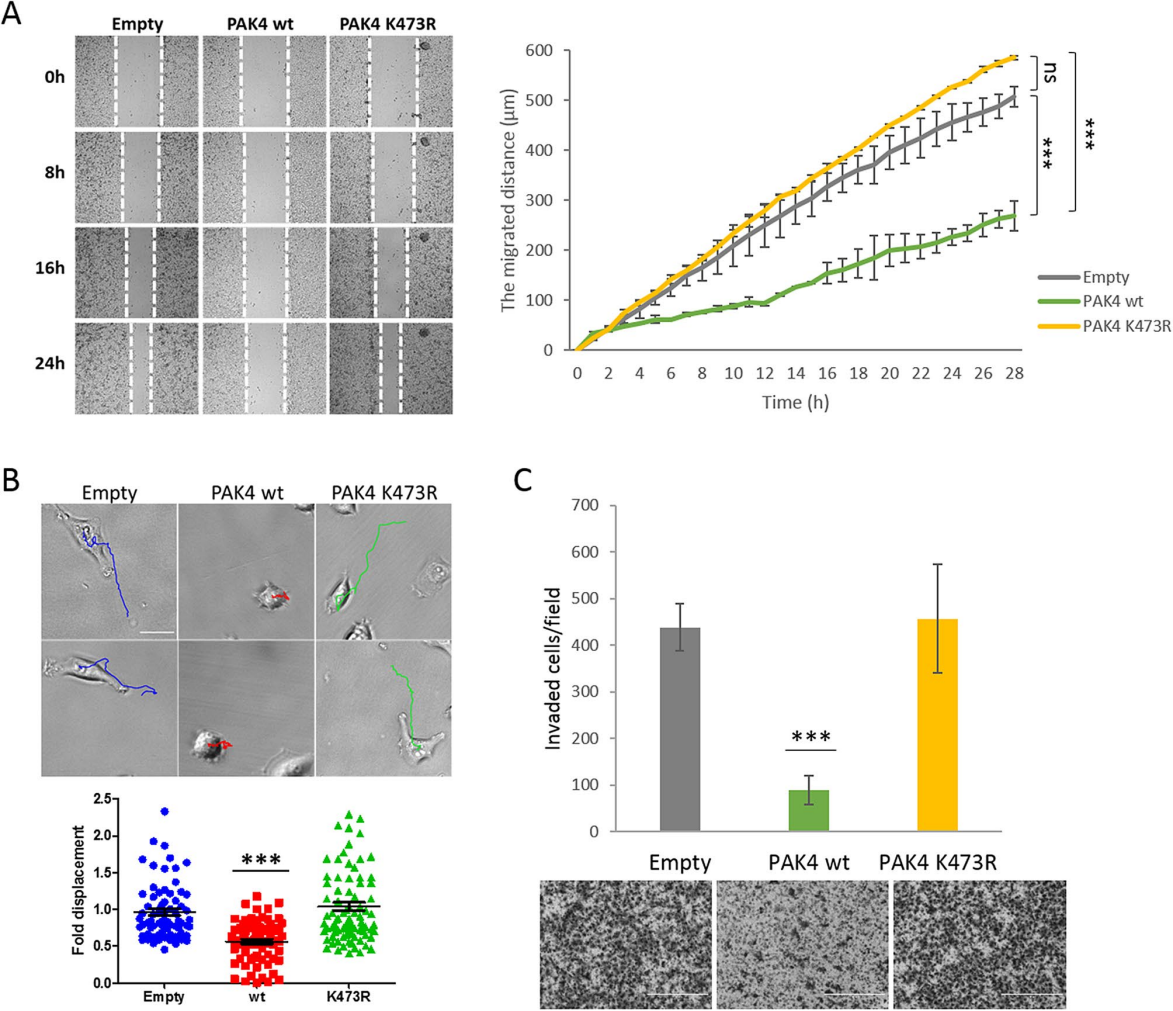
Unmethylated PAK4 promotes cell migration and cell invasion. (**A**) Migration assay. Confluent MDA-MB-231 cell cultures stably expressing empty plasmid, Flag PAK4 wild-type or Flag PAK4 K473R were serum starved before producing the scratch wound by dragging a 200 μl pipette tip across the layer of cells. Migration distance of the cells was monitored by a Lionheart™ FX Automated Microscope (4×). On the left, representative images of the cells at 0, 8, 16 and 24 h after producing the scratch, with white dashed lines indicating the wound borders. Error bars are s.d. Statistical analysis represents the last 21 time points (from 8 to 28 h of the experiment) using two-way ANOVA. ***p < 0.001, ns, not significant. Representative figure of 3 independent experiments. (**B**) Single cell migration assay. Approximately 5,000 MDA-MB-231 cells stably expressing empty plasmid, Flag PAK4 wild-type or Flag PAK4 K473R were plated and serum starved for over-night. Then, Hoechst stain was added and single cell motility was tracked for 4 h and analyzed by TrackMate (Fiji) software. The migration tracks of the cells are indicated in blue (empty), red (PAK4 wild-type) and green (PAK4 K473R). Scale bar 30 µm. The graph represents fold displacement of the cells relative to empty. Error bars are s.d. Statistical analysis was performed for n > 70 cells using one-way ANOVA. ***p < 0.001. The figure is accompanied with 6 movies in the supplementary data Movies S1-6***. ***(**C**) Invasion assay. MDA-MB-231 cells stably expressing empty plasmid, Flag PAK4 wild-type or Flag PAK4 K473R were serum starved, then added to the matrigel pre-coated inserts. After 24 h, invaded cells were stained with Dipp Kwik Differential Stain and visualized by EVOS FL Cell Imaging System and analyzed using Fiji software. The graph represents invaded cell counted per field. For each sample 5 fields were analyzed. Error bars are s.d. Statistical analysis was performed for 3 experimental repeats using one-way ANOVA. ***p < 0.001. Representative images of the invaded and stained cells at the bottom. Scale bar 400 µm.

Given the close connection between focal adhesions and cell migration^30,31^, we hypothesized that the changes in cell adhesion and focal adhesion formation might also affect cell migration. To test this, we monitored the migration of cells stably expressing empty, PAK4 wild-type or PAK4 K473R mutant in a wound healing assay, where a scratch is produced on a confluent monolayer of cells and the distance the cells made towards the gap is measured. Cells stably expressing PAK4 wild-type migrated significantly slower to close the gap than the PAK4 K473R or empty plasmid expressing cells (Fig. 6A). To get a better insight into the differences in the migration patterns of these cells, we examined the migration of single cells under the same experimental setup. The acquired images were stacked into time-lapse movies using Fiji^32^, and analyzed using TrackMate (a Fiji plugin^33^) to monitor cell movement. Calculated migration trajectories of empty and PAK4 K473R mutant cells exhibited significantly higher parameters of cell displacement, travel distance and straight-line speed of the cells, in comparison to PAK4 wild-type cells (Fig. 6B, Supplementary Fig. S7 and Movies S1-6). In contrast, cells stably expressing PAK4 wild-type travelled overall a shorter distance, moved a shorter distance from the start point to end point (displacement) and moved more slowly in a straight-line compare to empty or PAK4 K473R mutant cells. Interestingly, the morphology of the analyzed single cells visualized in Brightfield microscope, correlated well with actin structures and focal adhesion features of fixed cells shown in Figs. 3B, 4B and 5A. It seems that methylated PAK4 at K473—resulting in the impairment of focal adhesions and actin structures assembly—disrupts cell migration and therefore affects the velocity and the traveled distance of the cells (as observed in the single cell Movies S1-6). To verify that the differences are a result of cell migration and not cell proliferation, we tested the proliferation of the cells at the same conditions (Supplementary Fig. S8) and could not observe any significant difference between the different cell lines.

Migration is known as the movement of cells on 2D surfaces, however this characteristic is connected with cellular ability to move within 3D matrices, known as invasion. These migration findings led us to hypothesize that PAK4 K473 methylation by SETD6 affects cell invasion. Indeed, in a transwell invasion assay, where cells incubated on matrigel-coated transwell chambers were allowed to invade the membrane for 24 h, cells stably expressing empty or PAK4 K473R invaded the membrane, while PAK4 wild-type expressing cells presented impaired invasiveness (Fig. 6C). Altogether, our data support a model by which PAK4 methylation at K473 alters the generation of adhesion-related characteristics, such as filopodia and actin structures and focal adhesions. Methylated PAK4 interacts with paxillin and enhances paxillin phosphorylation at S272 which attenuates paxillin localization to the periphery of the cells and stimulates the disassembly of focal adhesions. Paxillin mislocalization leads to severe defects in cell adhesion, migration and invasion.

## Discussion

The serine/threonine kinase PAK4 has many roles in the regulation of diverse oncologically-related cellular pathways and phenotypes in several types of cancers such as; gastric^34^, prostate^35^, ovarian^36^, colon^37^ and breast ^38–40^. In most cases, the kinase activity of PAK4 and the phosphorylation of its downstream substrates is required to turn these pathways on and off^34–38^. Here we discovered that PAK4 cellular activity is controlled by lysine methylation in breast cancer cells. We show that SETD6-mediated PAK4 methylation at K473 stimulates the activation of Wnt/β-catenin target genes and inhibits cell adhesion and migration. Formation and stabilization of cell–matrix adhesion have a vital role in cell migration^41,42^ and they are important components of cell invasion during cancer progression^43^. Similarly, in our study, we show that loss of adhesion in cells stably expressing PAK4 wild-type correlates with poor ability of the cells to migrate both in a population and in single cell-based assays which result in reduced invasion.

Our data display a set of phenotypes partially overlapping those published before for PAK4. It was shown that PAK4 positively controls focal adhesions^8,27^ and downregulation of PAK4 leads to elevated adhesion to fibronectin and a decreased ability to migrate in breast cancer MDA-MB-231 cells^19^. In MCF-7 breast cancer cells, PAK4 expression increased migration of the cells on vitronectin, another extracellular matrix protein, and inhibited cell spreading and adhesion to vitronectin by stimulating integrin avβ5 and specifically phosphorylating integrin β5^44–46^. In addition, expression of a constitutively active PAK4 in fibroblast cells led to loss of actin structures and reduced cell adhesion by driving changes in cell spreading and rounding which contribute to oncogenic transformation^27^. The molecular mechanisms that govern these contradicting phenotypes, including the mRNA differential expression of β-catenin cell adhesion-related genes, remain unclear. However, one would speculate that redundant and compensating mechanisms exist to tightly regulate these delicate cellular functions. While our study focuses on PAK4 methylation in breast cancer cells, it is known that PAK4 expression is associated with promoting cell migration and invasion in other cancers as well^35,36,47^ and it remains an open question if PAK4 activity in these cancers is also regulated by SETD6.

We demonstrated that PAK4 methylation at K473 attenuates paxillin localization to focal adhesions as well as overall formation of filopodia structures, leading to reduced cell actin structures. Paxillin phosphorylation at S272 by PAK4 increases the turnover rate of focal adhesions^8^. Here, we show that the recruitment of paxillin to focal adhesions at the periphery of the cells depends on the methylation of PAK4 at K473. While the precise mechanism by which PAK4 methylation regulates paxillin phosphorylation and localization is still not fully clear, our data suggests that PAK4 methylation stabilizes the physical interaction with paxillin. One can speculate that this interaction may facilitate a direct or indirect recruitment of other proteins (methyl lysine binders) which stabilize the complex. The fact that PAK4 autophosphorylation occurs at the activation loop at S474^4^ which is adjacent to the K473, methylated by SETD6, can also provide an elegant methyl/phospho based mechanism to explain PAK4 effect on paxillin and presumably additional adhesion-related proteins.

In recent years, the link between lysine methylation and the regulation of oncogenically-related phenotypes such as adhesion, migration and invasion has been studied in depth in the context of histones^48–50^. However, the role of non-histone protein methylation is now emerging to be pivotal in the regulation of these processes. It was shown before that talin1, a cytoskeletal protein vital for cell adhesion and migration, is tri-methylated by the lysine methyltransferase Ezh2. Methylation of talin1 by Ezh2 leads to protein cleavage and to disruption of talin1 interaction with F-actin, leading to disassembly of focal complexes^51^. Additionally, loss of the methyltransferase Ezh2 in dendritic cells was reported to cause formation of large focal adhesions and impaired the migration properties of the cells^51^. It still remains to be determined if SETD6 and Ezh2 are regulating cell adhesion in a similar or distinct manner, but nevertheless, these findings highlight the importance of protein lysine methyltransferases and lysine methylation signaling in the regulation of cellular processes such as cell adhesion and migration.

## Materials and methods

### Cell lines, transfection and infection

Human embryonic kidney cells (HEK293T) and human breast adenocarcinoma cells (MDA-MB-231 and MCF-7) were maintained in Dulbecco’s modified Eagle’s medium (Sigma, D5671) with 10% fetal bovine serum (FBS) (Gibco), penicillin–streptomycin (Sigma, P0781), 2 mg/ml L-glutamine (Sigma, G7513) and non-essential amino acids (Sigma, M7145), at 37 °C in a humidified incubator with 5% CO_2_. Cell transfection was performed using polyethyleneimine (PEI) reagent (Polyscience Inc., 23966) according to the manufacturer’s instructions. CRISPR/CAS9-SETD6 knock-out cells were generated as described previously^13^. For stable transfection in MDA-MB-231 and MCF-7 cell lines, retroviruses were produced by transfecting HEK293T cells with pWZL-empty or pWZL-Flag-PAK4 wild-type, pWZL-Flag-PAK4 K473R, pWZL-Flag-SETD6 plasmids and with plasmids encoding VSV and gag-pol. Target cells were infected with the viral supernatants and selected with 650 or 600 μg/ml hygromycin B (TOKU-E), respectively.

### Plasmids

PAK4 and SETD6 sequences were amplified by PCR and were subcloned into pcDNA3.1 3×Flag and pcDNA3.1 3×HA plasmids. For viral infections, PAK4 and SETD6 were cloned into pWZL-Flag plasmid. For recombinant protein purification, PAK4 and SETD6 were cloned into pET-Sumo and pET-Duet plasmids, respectively. To generate PAK4 mutants, site-directed mutagenesis on PAK4 wild-type vector was performed using the following primers; PAK4 K31R: forward, 5′-GGGCTTCGACCAGCACGAGCAGCGCTTCACGGGGCTGCCCCGCC-3′, reverse, 5′-CGGGGCAGCCCCGTGAAGCGCTGCTCGTGCTGGTCGAAGCC-3′; PAK4 K51R: forward, 5′-GTCGGCTCGCCGGCCCCGCCCCCTCGTCGACCCCGCC-3′, reverse, 5′-GGCGGGGTCGACGAGGGGGCGGGGCCGGCGAGCCGACT-3′; PAK4 K78R: forward, 5′-CGGGGCAGCAAAGGTGCCCGCGATGGGGCCCTCACGCTGC-3′, reverse, 5′-CGTGAGGGCCCCATCGCGGGCACCTTTGCTGCCCCG-3′; PAK4 K442R: forward, 5′- GCGTCATCCACCGGGACATCAGGAGCGACTCGATCCTGCTGAC-3′, reverse, 5′-GTCAGCAGGATCGAGTCGCTCCTGATGTCCCGGTGGATGACGC-3′; PAK4 K473R: forward, 5′-GAAGTGCCCCGAAGGAGGTCGCTGGTCGGCACGCCCTAC-3′, reverse, 5′-CGTGCCGACCAGCGACCTCCTTCGGGGCACTTCCTTGC-3′; followed by DNA sequencing for confirmation. All PAK4 mutants were cloned into pET-Sumo plasmid. PAK4 K473R mutant was also cloned into pcDNA3.1 3×Flag and pWZL-Flag plasmids. PAK4 K350M construct was kindly provided by Audrey Minden (Rutgers University, USA) and Stefan Linder (University of Hamburg, Germany), and subcloned into pET-Sumo plasmid.

### Recombinant protein expression and purification

*Escherichia coli* Rosetta transformed with a plasmid expressing His- or His-Sumo tagged PAK4 wild-type, PAK4 mutant variants or SETD6 were grown in LB medium. Bacteria were harvested by centrifugation after IPTG induction and lysed by sonication on ice (25% amplitude, 1 min total, 10/5 s on/off). His-tagged proteins were purified using Ni–NTA beads (Pierce) or on a HisTrap column (GE) with the ÄKTA gel filtration system. Proteins were eluted by 0.5 M imidazole followed by dialysis to 10% glycerol in phosphate-buffered saline (PBS). Recombinant GST-SETD6 was expressed and purified as previously described^52^.

### In-vitro methylation assay

Methylation assay reactions contained 4 μg of His-Sumo-PAK4 wild-type or mutant PAK4 and 4 μg of His-SETD6 or GST-SETD6, 2 mCi of ^3^H-labeled *S*-adenosyl-methionine (Perkin- Elmer, AdoMet) and PKMT buffer (20 mM Tris–HCl, pH 8, 10% glycerol, 20 mM KCl, 5 mM MgCl_2_). The reaction tubes were incubated over-night at 30ºc. The reactions were resolved by SDS-PAGE for Coomassie staining (Expedeon, Instant*Blue*) or autoradiography.

### Antibodies, western blot analysis and immunoprecipitation

Primary antibodies used were: anti-Flag (Sigma, F1804), anti-HA (Millipore, 05–904), anti-actin (Abcam, ab3280), anti-pan-methyl (Cell signaling, 14679), anti-PAK4 (Cell Signaling, 3242), anti-β-catenin (Abcam, ab32572), anti-non-phospho (active) β-catenin (Cell signaling, 8814), anti-phospho-β-catenin S675 (Cell Signaling, 4176), anti-SETD6 (Genetex, GTX629891), paxillin S272-ph (Biorbyt, orb128603) and anti-histone3 (H3) (Abcam, ab10799). Anti-paxillin (BD Biosciences, 610052) antibody was kindly provided by Ronen Zaidel-Bar from Tel-Aviv University Medical School, Israel. HRP-conjugated secondary antibodies, goat anti-rabbit and goat anti-mouse were purchased from Jackson ImmunoResearch (111-035-144 and 115-035-062, respectively). Fluorescently labeled secondary antibodies: Alexa 647 anti-rabbit (Invitrogen, A-21443), Alexa 594 anti-rabbit (Abcam, ab150080) and Alexa 568 anti-mouse (Invitrogen, A-11061). For Western blot analysis, cells were homogenized and lysed in RIPA buffer (50 mM Tris–HCl, pH 8, 150 mM NaCl, 1% Nonidet P-40, 0.5% sodium deoxycholate, 0.1% SDS, 1 mM DTT, and 1:100 protease inhibitor mixture (Sigma)). Samples were resolved on SDS-PAGE, followed by Western blot analysis. For immunoprecipitation, proteins extracted from cells were incubated over-night at 4ºc with FLAG-M2 beads (Sigma, A2220). The beads were then washed three times with RIPA buffer and submitted to Western blot analysis.

### Flow cytometry (FACS analysis)

MDA-MB-231 cells stably expressing Flag PAK4 wild-type or Flag PAK4 K473R were fixed, permeabilized and immunostained using anti-non-phospho (active) β-catenin antibody and Alexa Fluor 647 conjugated-secondary antibody according to the manufacturer’s instructions (Cell signaling, flow cytometry protocol). Then, 5 × 10^4^ cells were tracked for flow cytometry analysis (Guava easyCyte Flow Cytometer).

### Chromatin extraction

To extract chromatin fraction, biochemical fractionation was performed as previously described^53^, with the addition of another step. In brief, the chromatin pellet was resuspended in RIPA buffer including 1 mM MgCl_2_ and benzonase nuclease enzyme (Sigma) and incubated on ice for 30 min. The supernatant was collected by low-speed centrifugation (5 min, 1700 × *g*) at 4 °C. The soluble chromatin was resolved by SDS-PAGE gel and analyzed by Western blot.

### RNA extraction and real-time qPCR

Total RNA was extracted using the NucleoSpin RNA Kit (Macherey–Nagel). 200 ng of the extracted RNA was reverse transcribed to cDNA using the iScript cDNA Synthesis Kit (Bio-Rad) according to the manufacturer’s instructions. Real-time qPCR was performed using the UPL probe library system (Roche) in a LightCycler 480 System (Roche). The real-time qPCR primers were the following;

GAPDH: forward, 5′-AGCCACATCGCTCAGACAC-3′,

Reverse, 5′-GCCCAATACGACCAAATCC-3′;

CTBP1: forward, 5′-CGAGTCGGAACCCTTCAG-3′,

Reverse, 5′-CAGATGAGGTTGGGTGCAT-3′;

JUN: forward, 5′-CCAAAGGATAGTGCGATGTTT-3′,

Reverse, 5′-CTGTCCCTCTCCACTGCAAC-3′;

MET: forward, 5′-TCGATCAGGACCATCAACC-3′,

Reverse, 5′-TCAATGGGATCTTCGTGATCT-3′;

SENP2: forward, 5′-TCACTGGCTCAATGATGAAGTC-3′,

Reverse, 5′-AGTGCTGGATAGCCTTGCTT-3′;

SOX9: forward, 5′-GTACCCGCACTTGCACAAC-3′,

Reverse, 5′-TCTCGCTCTCGTTCAGAAGTC-3′;

NRCAM: forward, 5′-CGAGTGGTCAATGGGAAAG-3′,

Reverse, 5′-TTCAAAGACGAGGGAGCACT-3′;

FN1: forward, 5′-GGGAGAATAAGCTGTACCATCG-3′,

Reverse, 5′-TCCATTACCAAGACACACACACT-3′;

LAMC2: forward, 5′-CTACTTCGGGGACCCATTG-3′,

Reverse, 5′-GGTTACAGTTGCAAGCTCGAC-3′;

NEDD9: forward, 5′-TGTGGATCCAAGCTCTGATAGTT-3′,

Reverse, 5′-TGTGGTCCTGGCCTCTAAAC-3′;

IGFBP7: forward, 5′-ACTGGCTGGGTGCTGGTA-3′,

Reverse, 5′-TGGATGCATGGCACTCATA-3′;

L1CAM: forward, 5′-AGTGCCAGTCGAACACCAG-3′,

Reverse, 5′-GAAGGACAGTTGCCCCTTG-3′;

Gene expression levels were normalized relative to GAPDH gene and controls of the experiment.

### Wash assay

Cells were plated in a 24-well plate to reach 95–100% confluency the following day. Cells were then serum starved (2% FBS) over-night, washed with PBS, and finally fixed and stained with crystal violet solution (0.5% crystal violet and 20% methanol).

### Fibronectin adhesion assay

For cell adhesion assay to fibronectin, cells were serum starved (0.5% FBS) over-night. Then, cells were harvested and 3 × 10^5^ (MDA-MB-231) cells/well were plated on a fibronectin (Millipore, 341631) pre-coated 96-well plate (2.5 µg/well) for 3.5 h, followed by a PBS wash and crystal violet staining (0.5% crystal violet and 20% methanol). Crystal violet staining was solubilized in 2% SDS and quantified at 550 nm using Tecan Infinite M200 plate reader.

### Cell migration

For cell migration (wound healing assay), cells were serum starved (0.5% FBS) over-night, then 3.5 × 10^5^ cell/well were plated on fibronectin pre-coated 48-well plate. After 3.5 h, the scratch wound was produced by dragging a 200 μl pipette tip across the layer of cells. The migrated distance of the cells towards the gap was monitored by a Lionheart^TM^ FX Automated Microscope (4×).

For single cell migration assay, 5,000 cells/well were plated (in a 48-well plate), serum starved (0.5% FBS) for over-night, then the media was changed to complete media with Hoechst stain. Cell movement was monitored every 5 min for 4 h by Lionheart^TM^ FX Automated Microscope (20×) and analyzed using TrackMate (Fiji) software^33^.

### Cell proliferation

For the cell proliferation assay, cells were serum starved (0.5% FBS) over-night, then 7.5 × 10^3^ cell/well were plated on a fibronectin pre-coated 96-well plate. Cell proliferation was monitored by a Lionheart™ FX Automated Microscope (4×) every 2 h.

### Microscopy

Cells were plated on cover slips, serum starved (0.5% FBS) over-night, stained with Vybrant^TM^ DiI Cell-Labeling Solution to stain cell membranes (Invitrogen, V22885), then fixed with 2% PFA and stained with Hoechst dye. For phalloidin staining of F-Actin (Invitrogen, O7466), cells were fixed with 4% PFA, triton X-100 permeabilized and stained with phalloidin, then mounted on slides using fluoromount containing DAPI dye (SouthernBiotech, 0100-20). For paxillin or paxillin S272-ph staining, following fixation and permeabilization, cover slips were incubated with anti-paxillin or paxillin S272-ph antibody for 3–6 h, then incubated with phalloidin and Alexa Fluor 568 or Alexa Fluor 594 conjugated-secondary antibody for 1 h, then mounted using fluoromount containing DAPI dye.

Finally, stained cells were visualized by confocal laser microscope ZEISS LSM880 Airyscan using the Plan Apochromat 63×/1.4 oil DIC M27 or Plan Apochromat 40×/1.4 Oil DIC M27 objectives^54^. Percent cells with filopodia or actin structures were manually counted. Paxillin area size was analyzed using the "YEN" threshold tool in the Fiji software^32^. For the quantification of paxillin S272-ph intensity, all images were converted to 8-bit grayscale. Following which, cell borders were traced using the wand tracing tool in Fiji and integral fluorescence density for corresponding channel was calculated within the defined area and normalized to the integral fluorescence density of DAPI staining obtained in the same area.

### Invasion assay

The invasion assay was performed using ThinCerts inserts, chambers with 8 µm pore membranes (Greiner bio-one, 662638). The inserts were coated with ~ 30 µg Matrigel (Corning Matrigel Basement Membrane Matrix, Phenol Red-free, *LDEV-free, Product Number 356237) and dried for 2 h at 37 °C. Over-night serum starved cells were harvested and 5 × 10^4^ cells in 0.5% FBS DMEM were added to the upper side of the inserts, and the wells were filled with 10% FBS DMEM. After 24 h, the non-invasive cells on the upper side of the inserts were removed using cotton swabs. The invaded cells were fixed and stained using the Dipp Kwik Differential Stain kit (American Mastertech Scientific). The stained cells were imaged by EVOS FL Cell Imaging System (Thermo Fisher Scientific) using 10 × objective. The images were analyzed using Fiji software^32^.

### Statistical analysis

Statistical analyses for all assays were analyzed with GraphPad Prism software, using Student’s two-tailed t-test (unpaired), one-way analysis of variance (ANOVA) with a Tukey's post-test or two-way ANOVA followed by Bonferroni's post-test.

## Supplementary information

Supplementary file1

Supplementary file2

Supplementary file3

Supplementary file4

Supplementary file5

Supplementary file6

Supplementary file7
