# Supplementary material for: PAK4 methylation by the methyltransferase SETD6 attenuates cell adhesion
Source: Sci Rep. 2020 Oct 13;10:17068. doi: 10.1038/s41598-020-74081-1 (PMC7555502; doi:10.1038/s41598-020-74081-1)
Supplement: Supplementary file 7 — Supplementary file7 [file 41598_2020_74081_MOESM7_ESM.pdf]

**PAK4 methylation by the methyltransferase SETD6 attenuates cell adhesion**

Zlata Vershinin[1,2*], Michal Feldman[1,2*], and Dan Levy[1,2#]

[1]The Shraga Segal Department of Microbiology, Immunology and Genetics and the

[2]National Institute for Biotechnology in the Negev, Ben-Gurion University of the Negev,

P.O.B. 653, Be'er-Sheva 84105, Israel

[#]Correspondence should be addressed to D.L. (ledan@post.bgu.ac.il)

[*]Equal contribution

# Supplementary Information

**Figure S1. Conserved lysine residues and methylation assays of different PAK4 lysine mutants**. ***A,*** A multiple alignment of conserved lysine residues of PAK4 in different organisms. Multiple alignment was performed using COBALT tool[55] for *Homo sapiens*, *Mus musculus*, *Danio rerio* and *Drosophila melanogaster* PAK4 protein sequences. Chosen lysine residues are indicated with arrows. ***B,*** *In-vitro* methylation assay. *From left to right: (1)* Recombinant His-Sumo-PAK4 wild-type, the His-Sumo-PAK4 K31R mutant or His-Sumo-PAK4 K51R mutant were incubated with His-SETD6. *(2)* Recombinant His-Sumo-PAK4 wild-type or the His-Sumo-PAK4 K350M mutant were incubated with or without GST-SETD6. *(3)* Recombinant His-Sumo-PAK4 wild-type, the His-Sumo-PAK4 K78R mutant or His-Sumo-PAK4 K442R mutant were incubated with or without His-SETD6. All reactions also contained $^{3}$H-labeled SAM. Proteins were then subjected to SDS-PAGE followed by exposure to autoradiogram to detect $^{3}$H-labeled proteins or Coomassie staining to detect all proteins. Uncropped gels are shown in Supplementary Fig. S10.

**Figure S2. Methylation of PAK4 at K473 upregulates the expression of activated β-catenin in cells.** ***A,*** MDA-MB-231 stably expressing Flag PAK4 wild-type or Flag PAK4 K473R cells were fixed, permeabilized and immunostained with non-phospho (active) β-catenin antibody and then with Alexa Fluor 647 conjugated-secondary antibody for FACS analysis. Histograms represent an overlay of active (non-phospho) β-catenin expression in PAK4 wild-type (green) and PAK4 K473R cells (grey). ***B,*** MDA-MB-231 cells stably expressing Flag PAK4 wild-type or Flag PAK4 K473R were subjected to chromatin isolation using a biochemical fractionation protocol followed by Western blot analysis to determine PAK4, β-catenin and active β-catenin protein levels. H3 signal served as a loading control. Uncropped gels are shown in Supplementary Fig. S10.

**Figure S3. PAK4 K473me does not alter β-catenin protein levels and the activation Wnt/β-catenin target genes in MCF-7 cells**. ***A,*** Cell extracts of MCF-7 cells stably expressing empty plasmid, Flag PAK4 wild-type or Flag PAK4 K473R were submitted to Western blot to detect endogenous protein levels of β-catenin, active (non-phospho) β-catenin and β-catenin S675-ph. Uncropped gels are shown in Supplementary Fig. S10. ***B,*** mRNA was extracted from MCF-7 cells stably expressing empty plasmid, Flag PAK4 wild-type or Flag PAK4 K473R. Transcript levels of the indicated Wnt/β-catenin target genes were determined by qPCR. mRNA levels were normalized to GAPDH and then to empty cells. Error bars are s.e.m. Statistical analysis was performed for 3 experimental repeats using one-way ANOVA. $*p < 0.05$, $**p < 0.01$, $***p < 0.001$.

**Figure S4. PAK4 K473me decreases cell adhesion**. Wash assay. Confluent MDA-MB-231 cells stably expressing empty plasmid, Flag PAK4 wild-type or Flag PAK4 K473R, and MDA-MB-231 CRISPR SETD6 knock-out (SETD6 KO) cells were serum starved over-night, washed with PBS, then cells were fixed and stained with crystal violet solution.

**Figure S5. SETD6 rescues Wnt/β-catenin target genes activation and adhesion-related phenotypes**. ***A,*** MDA-MB-231 CRISPR SETD6 knock-out cells stably expressing Flag PAK4 wild-type or Flag PAK4 K473R mutant, with or without Flag SETD6 were subjected to Western blot using the indicated antibodies. Uncropped gels are shown in Supplementary Fig. S10. ***B,*** MDA-MB-231 CRISPR SETD6 knock-out (SETD6 KO) cells stably expressing Flag PAK4 wild-type with empty or Flag SETD6, were fixed and stained with Vybrant™ DiI Cell-Labeling Solution and Hoechst stain and visualized the cells by a confocal microscope (40x). Scale bar 10 µm. Graph on the right represents percent cells with filopodia structures manually counted. Error bars are s.d. Statistical analysis was performed for n > 52 cells per condition

using Student's t-test. ***$p < 0.001$. ***C,*** mRNA was extracted from MDA-MB-231 CRISPR SETD6 knock-out cells stably expressing Flag PAK4 wild-type or Flag PAK4 K473R mutant, with or without Flag SETD6. Transcript levels of the indicated Wnt/β-catenin target genes were determined by qPCR. mRNA levels were normalized to GAPDH and then to empty cells. Error bars are s.e.m. Statistical analysis was performed for 3 experimental repeats using one-way ANOVA. *$p < 0.05$, **$p < 0.01$, ***$p < 0.001$.

**Figure S6. Methylation of PAK4 at K473 upregulates paxillin phosphorylation at S272.** ***A,*** MDA-MB-231 cells stably expressing empty plasmid, Flag PAK4 wild-type or Flag PAK4 K473R were fixed and stained with phalloidin (green), paxillin S272-ph (red) and DAPI. Cells were visualized by a confocal microscope (63x). Scale bar 10 µm. The graph represents the paxillin S272-ph (p272) signal intensity normalized to DAPI intensity. Error bars are s.d. Statistical analysis was performed for $n \geq 6$ cells per condition using one-way ANOVA. **$p < 0.01$. ***B,*** Cell extracts of MDA-MB-231 cells stably expressing empty plasmid, Flag PAK4 wild-type or Flag PAK4 K473R were submitted to Western blot to determine paxillin protein levels. Uncropped gels are shown in Supplementary Fig. S10.

**Figure S7. Unmethylated PAK4 promotes single cell migration.** Additional parameters measured in a single cell migration assay as described in Figure 6B. On the left, graph presenting the straight-line speed of the cells relative to empty. On the right, graph presenting the travelled distance of the cells relative to empty. Error bars are s.d. Statistical analysis was performed for $n > 70$ cells using one-way ANOVA. ***$p < 0.001$.

**Figure S8. PAK4 methylation at K473 does not affect cells proliferation.** Proliferation assay. MDA-MB-231 cell cultures stably expressing empty plasmid, Flag PAK4 wild-type or

Flag PAK4 K473R were serum starved over-night. Cells were then plated, and cell proliferation was monitored by a Lionheart™ FX Automated Microscope (4x) every 2 h. Error bars are s.d. Statistical analysis was performed for 4 experimental repeats using two-way ANOVA. ns, not significant.

**Figure S9. Supporting uncropped gels.** Uncropped gels of the experiments shown in figures 1B, 1C, 2A and 5B.

**Figure S10. Supporting uncropped gels.** Uncropped gels of the experiments shown in supplementary figures S1B, S2B, S3A, S5A and S6B.

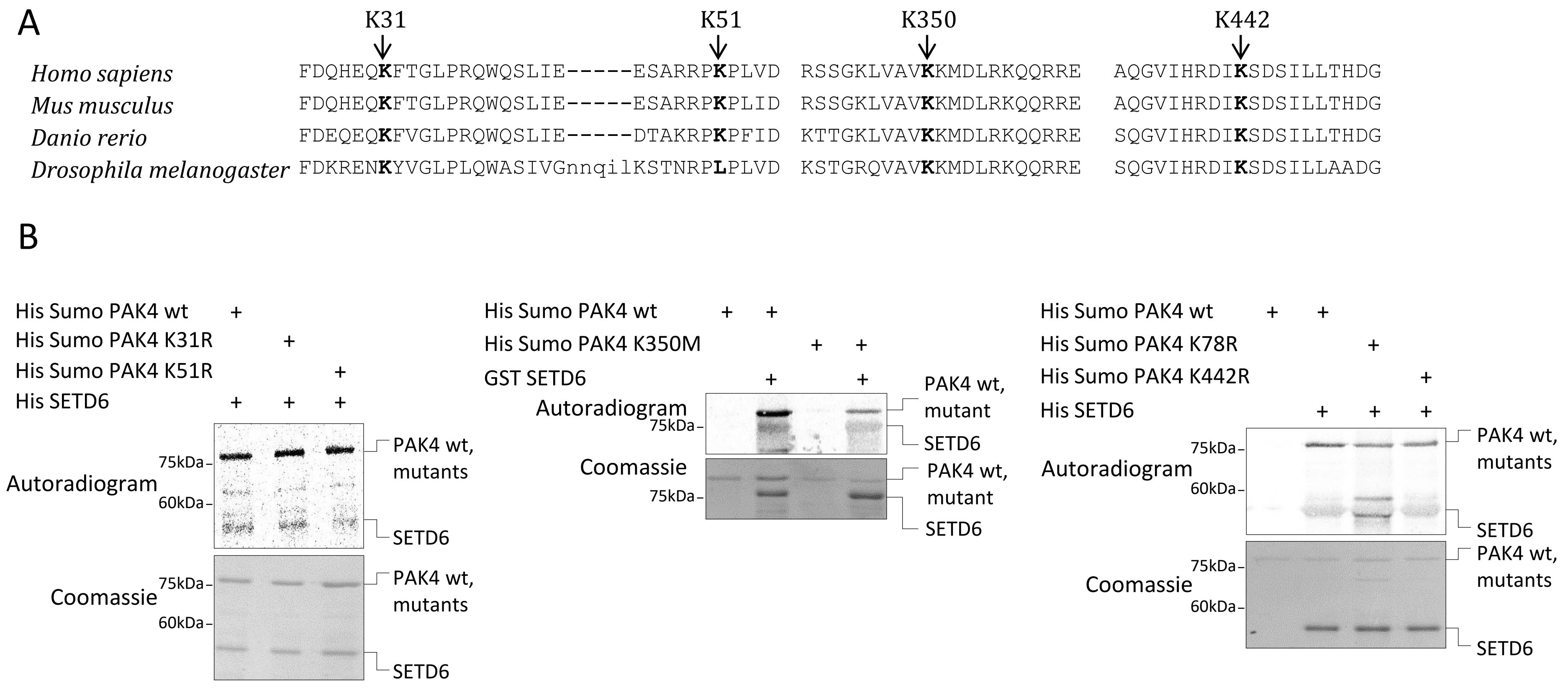


# Supplementary Figure S1

A

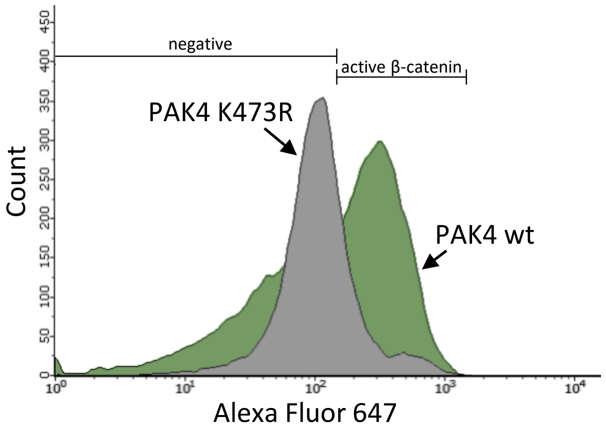


B

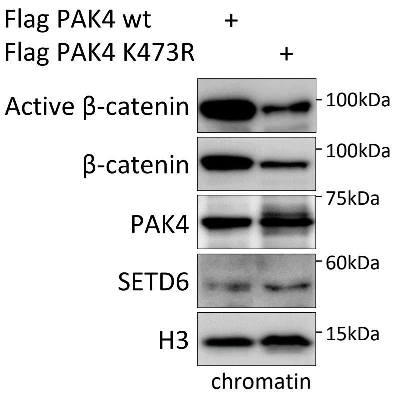


**Supplementary Figure S2**

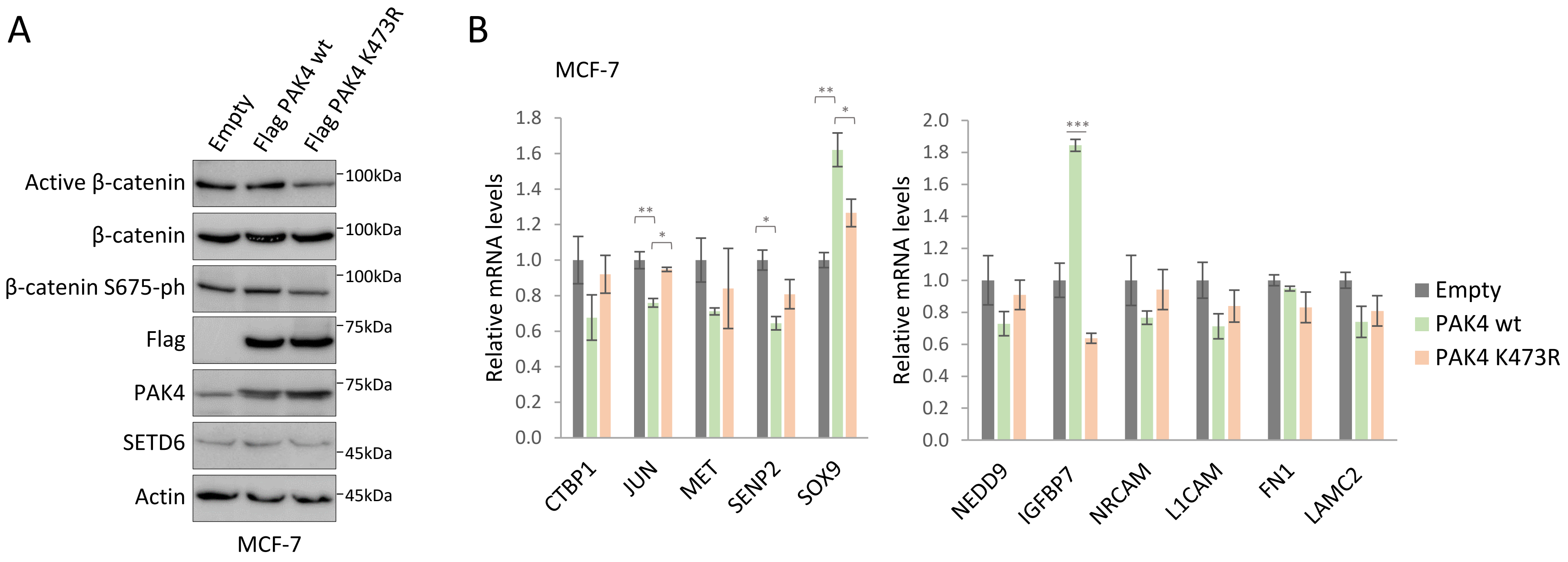


**Supplementary Figure S3**

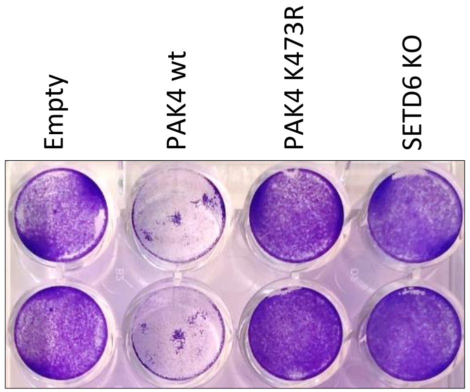


**Supplementary Figure S4**

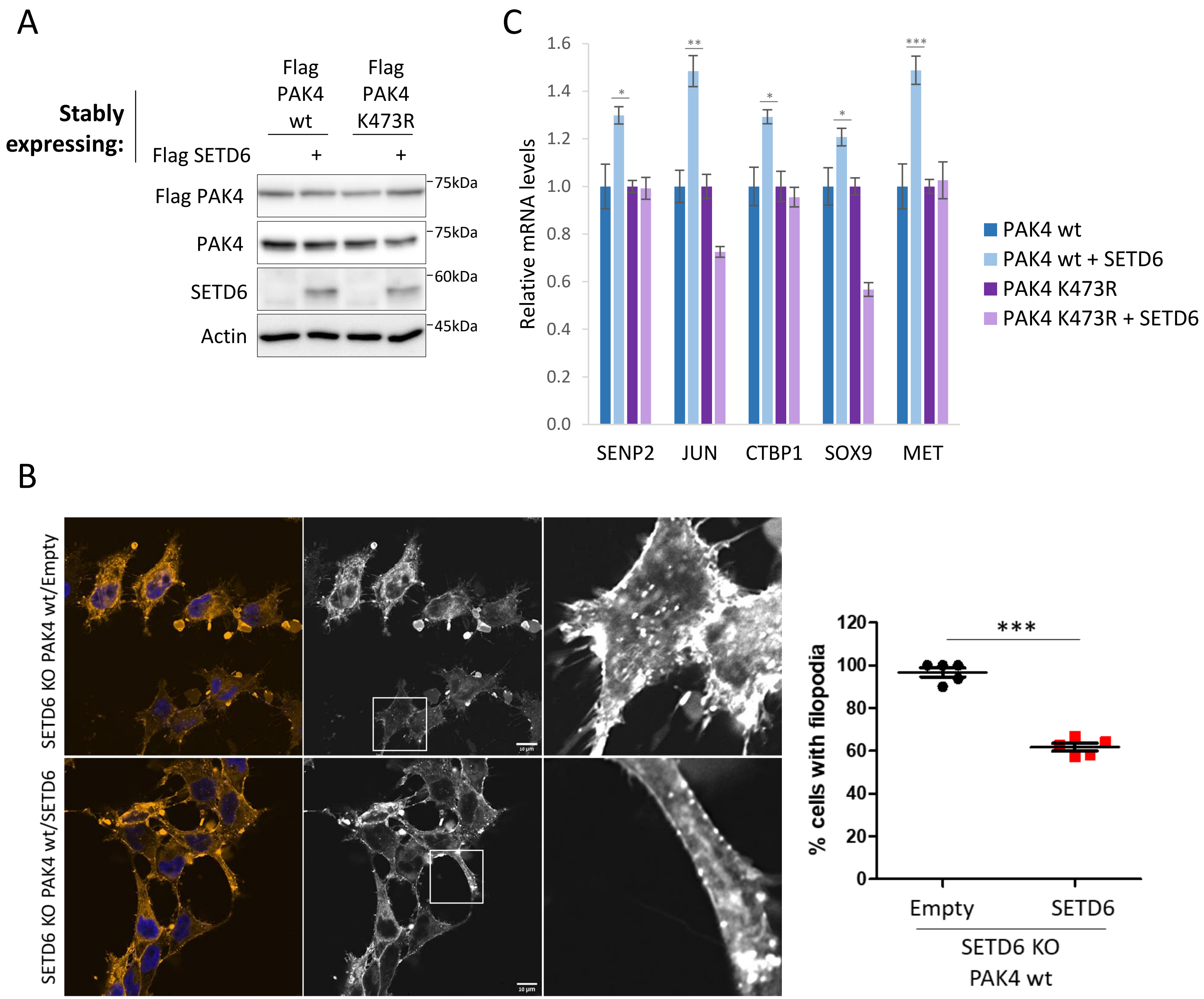


**Supplementary Figure S5**

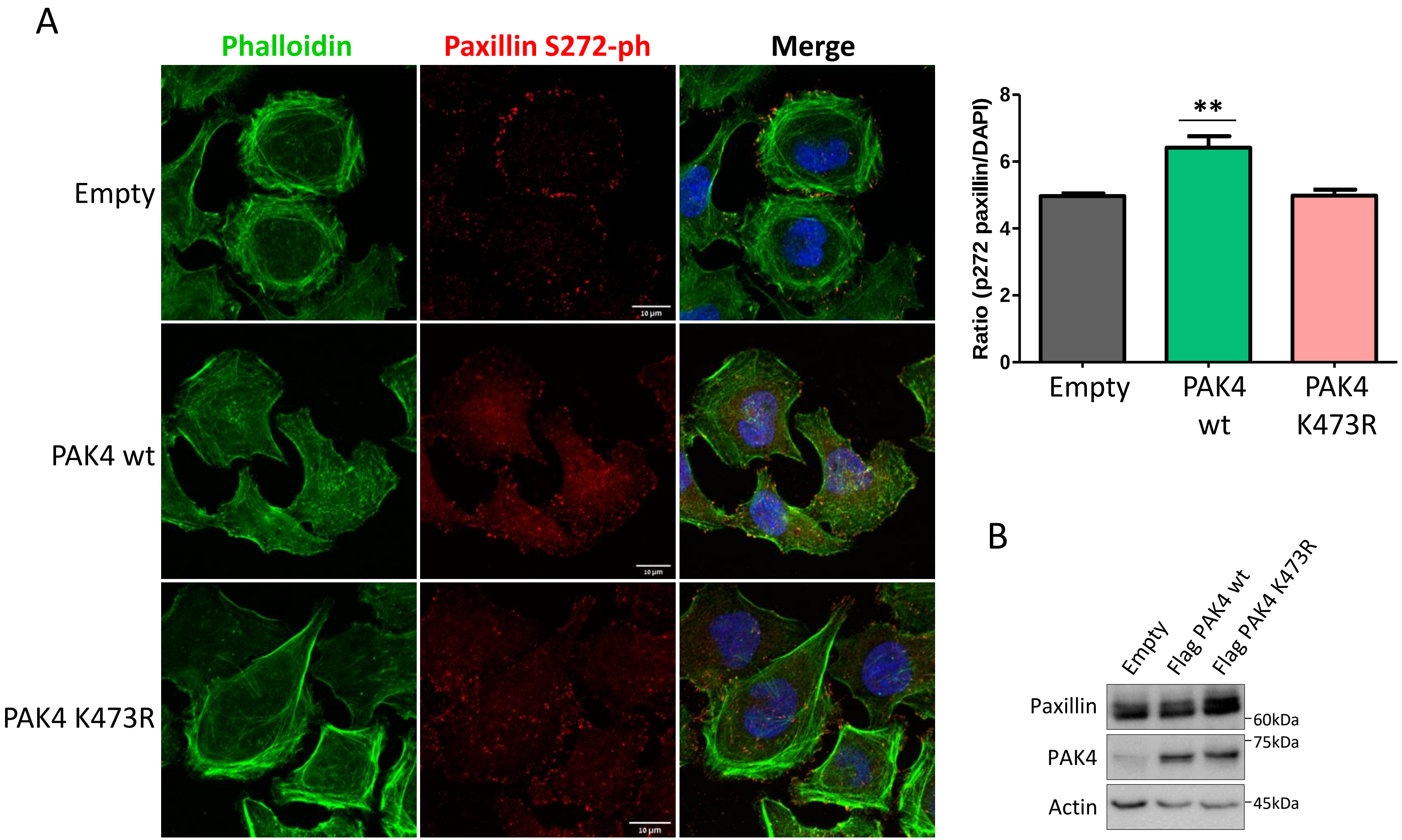


**Supplementary Figure S6**

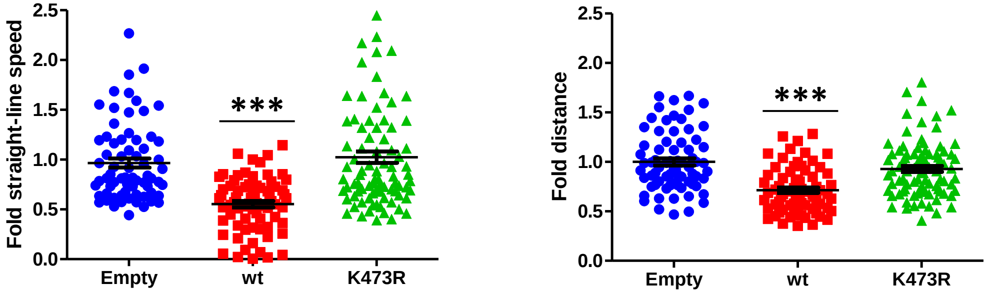


# Supplementary Figure S7

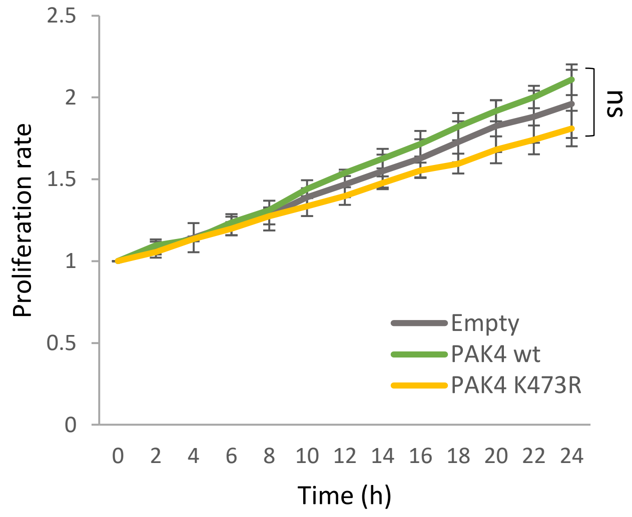


**Supplementary Figure S8**

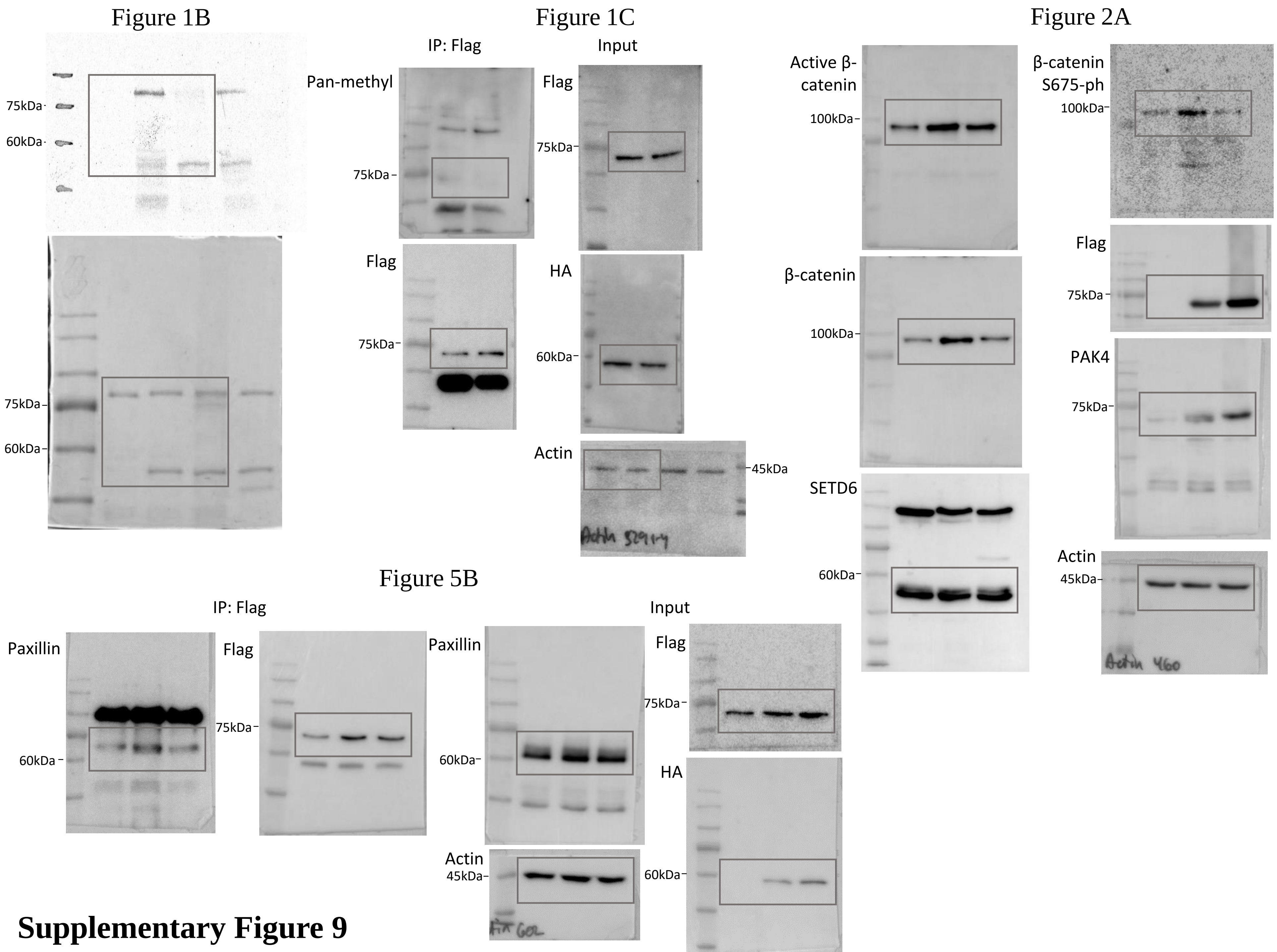


**Supplementary Figure 9**

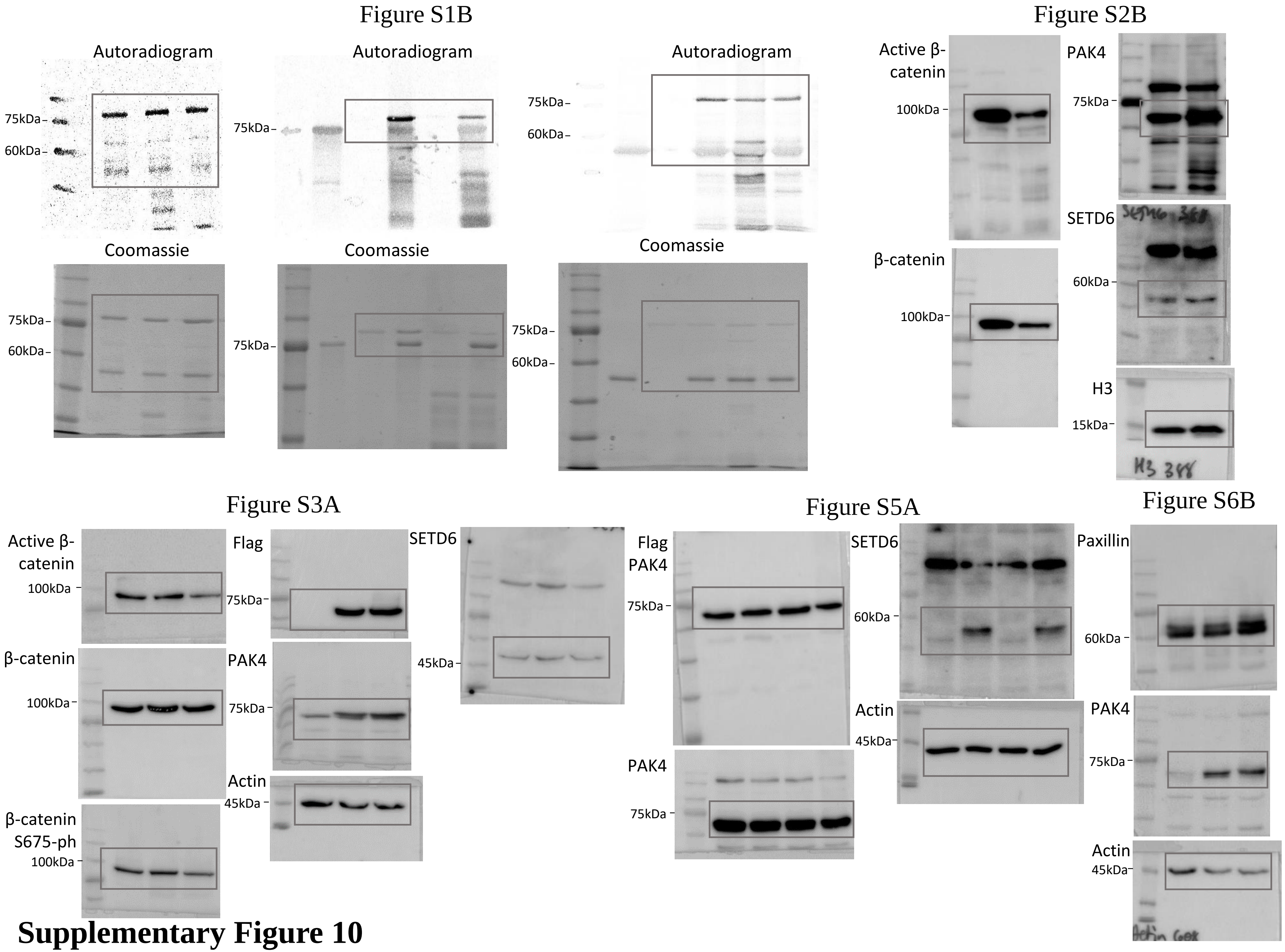


**Supplementary Figure 10**
